# Four new species of the genus *Delia* Robineau-Desvoidy in the Yunnan Province of China (Diptera, Anthomyiidae)

**DOI:** 10.3897/zookeys.693.12965

**Published:** 2017-08-24

**Authors:** Jing Du, Wanqi Xue

**Affiliations:** 1 College of Life Science, Shenyang Normal University, Shenyang 110034, China; 2 Institute of Entomology, Shenyang Normal University, Shenyang 110034, China

**Keywords:** Anthomyiidae, *Delia*, Diptera, new species, Yunnan

## Abstract

Four new species of the genus *Delia* from Yunnan Province are described: *Delia dentiaedeagus* **sp. n.**, *Delia longiabdomina* **sp. n.**, *Delia nigerihalteres* **sp. n.**, and *Delia tuberisurstyla* **sp. n.** A catalogue of all *Delia* species recorded from Yunnan Province has also been included.

## Introduction

*Delia* is one of the largest genera of Anthomyiidae (Diptera), established by Robineau-Desvoidy (1830). The type species *Delia floricola* Robineau-Desvoidy, 1830 was designated by Coquillett (1910). It can be recognized by the following combination of characteristics: eyes usually bare; frons usually narrow in male and broad in female; with pairs of inter frontal setae; legs black or yellow; fore tibia with 0–1 *ad*, 1–2 medial *p* or 1–2 *pv* and 1 apical *pv*; mid tibia with 1 *pd*; hind femur without *pv* row or apical *pv*; surstylus longer than cercus, not bifurcate apically; aedeagus slender, apical part with paraphallus in most species (Ackland 1967 and 2008, Griffiths 1991, Dely-Draskovits 1993, Fan et al. 1988, Fan and Zheng 1993, Hennig 1974, Suwa 1977, Wei et al. 1996, Xue and Zhang 1996, Xue and Du 2008, and 2009).

There are 103 species distributed in China, among which 12 are located in Yunnan Province. Yunnan is one of the most diverse places in China: its complex and varied terrains and landforms as well as the different climates have created a natural paradise for biodiversity. After several collecting periods, four new species were found. This paper includes a complete report on these new species and a full catalogue of all species distributed in Yunnan Province.

## Materials and methods

All specimens were collected from Yunnan Province of China. Type specimens are deposited in the Diptera collection of the Institute of Entomology, Shenyang Normal University (**IESNU**).

### Abbreviations used in the descriptions:

***a*** anterior setae;

***acr*** acrostichal setae;

***ad*** anterodorsal setae;

***av*** anteroventral setae;

***d*** dorsal setae;

***dc*** dorsocentral setae;

***ial*** intra-alar setae;

***p*** posterior setae;

***pd*** posterodorsal setae;

***post acr*** postsutural acrostichal setae;

***post dc*** postsutural dorsocentral setae;

***pra*** prealar setae;

***prst acr*** presutural acrostichal setae;

***prst dc*** presutural dorsocentral setae;

***pv*** posteroventral setae; and

**R_4+5_** branch of radius.

## Taxonomy

### Delia dentiaedeagus sp. n.

Taxon classificationAnimaliaDipteraAnthomyiidae

http://zoobank.org/8F7E8B49-7023-4191-856C-32CBAF7EE7C6

Figure 1

#### Type material.

***Holotype*.** China, Yunnan Province, Baimang Snowberg, 4000 m, 29 May 2007, Dandan Wang Co., ♂(IESNU). ***Paratype*.** China, Yunnan Province, Baimang Snowberg, 4000 m, 31 May 2007, Lu Zhang Co., 2♂♂(IESNU).

#### Diagnosis.

Arista short ciliated, longest hairs shorter than its basal diameter; legs black; mid tarsomere 1 with long *ad*; processes of 5th sternite slender, with long and dense setae, without spine or protrusion.

**Figure 1.**
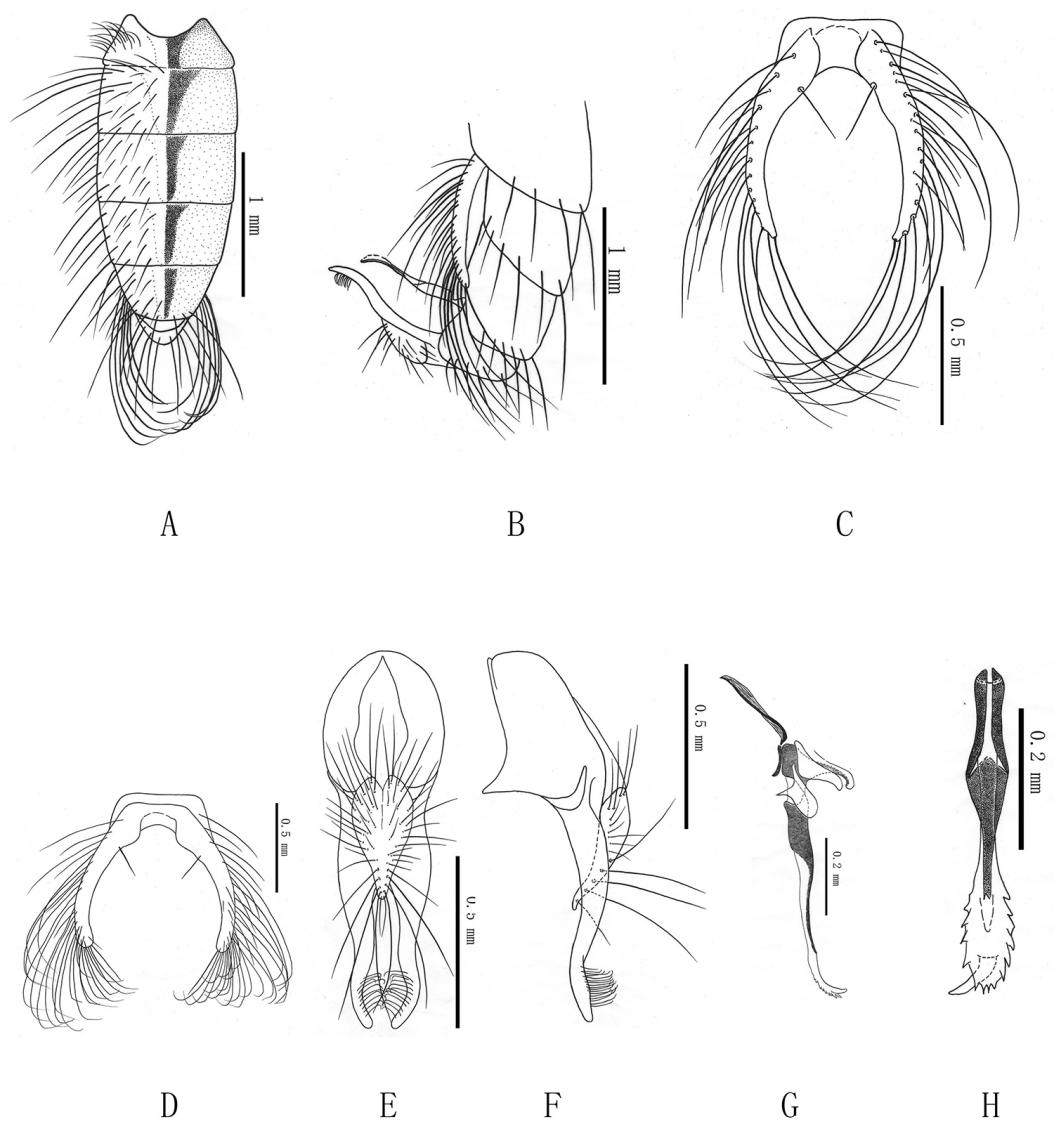
***Delia dentiaedeagus* sp. n. A** male, abdomen in dorsal view **B** male, distal half of abdomen in profile **C** male, 5th sternite in ventral view, in holotype **D** male, 5th sternite in ventral view, in paratype **E** male, terminalia in posterior view **F** male, terminalia in profile **G** male, aedeagus in profile **H** male, distal part of aedeagus in anterior view.

#### Description.

**Holotype Male.** Body length 4.5–5.0 mm.

*Head*. Eye bare; frontal vitta dark brown without interfrontal setule; fronto-orbital plate adjacent; frons as wide as anterior ocellus; without orbital setae; fronto-orbital plate and parafacial with grey dust; 5–6 pairs of frontal setae, situated on lower half of frons; parafacial subequal to the width of antenna; antenna black, postpedicel approx. 2.2–2.5 times longer than broad; arista short ciliated, longest hairs shorter than its basal diameter; lower facial margin not projecting, situated behind frontal angle in profile; genal height approx. 1/5 eye height; anterior margin of gena with one row of upcurved subvibrissal setulae; prementum black, without dust, approx. 3.5 times longer than broad; palpus black, slightly shorter than prementum.

*Thorax*. Black in ground colour, with grey to brownish grey dust; scutum with three black vittae, the middle one extended to scutellar suture; *prst acr* two rows, only one pair of *post acr* developed which are placed in front of scutellum, *dc* 2+3, *ial* 0+2; notopleural despression bare apart from two strong setae; *pra* as long as posterior notopleural; scutellum bare on disc centrally and basally; katepisternals 1+2.

*Wing*. Slightly transparent. Vein fuscous and basicosta black; Costa setulous near its base on ventral surface only; costal spine distinct; radial node bare; squamae brownish yellow, lower squama not projecting, approx. 2/3 length of upper; haltere yellowish.

*Legs*. Entirely black; fore tibia with one medial *p*; mid femur without distinct *av* row, a row of seta-like *pv* in basal half, only two setae in basal developed; mid tibia without *av* or *ad*, two *pd* and two *pv*; mid tarsomere 1 with distinct *ad*; hind femur with 2–3 *av* and 2–3 *pv* in distal part; hind tibia with four *av*, three *ad*, three *pd*, and 5–6 *pv* in middle part, without apical *pv*; hind tarsi longer than tibiae, all claws and pulvilli normal, slightly shorter than tarsomere 5.

*Abdomen*. Black, rhombic in dorsal view, with grey to brownish dusting; all tergites with black vittae in middle part; dorsal setae sparse, lateral setae long; 6^th^ tergite bare; 1^st^ sternite with setae.

**Female.** Unknown.

#### Remarks.

This new species is similar to *Delia felsicanalis* Fan & Wu in Fan et al., 1984, but differs from it in the following features: male frontal vitta dark brown; fronto-orbital plate adjacent; parafacial subequal to the width of antenna; prementum 3.5 times longer than broad; basicosta black; haltere yellowish; hind tibia with four *av*, three *ad* and 5–6 *pv* in middle part.

#### Etymology.

The species name is derived from the Latin words “*dent*” meaning tooth and “*aedeagus*” meaning aedeagus, referring to its apical aedeagus with tooth.

#### Distribution.

China, Yunnan Province (Baimang Snowberg).

### Delia longiabdomina sp. n.

Taxon classificationAnimaliaDipteraAnthomyiidae

http://zoobank.org/F72356C7-A54B-4721-B802-A08723D0114B

Figure 2

#### Type material.

***Holotype*.** China, Yunnan Province, Yulong Snowberg, 4506 m, 29 June 2006, Baifeng Wang Co., ♂ (IESNU). *Paratypes*. China, same data as holotype, 1 ♀ and 4♂♂(IESNU); China, Yunnan Province, Yulong Snowberg, 4506–4571 m, 29 June 2006, You Wang Co., 5♂♂ (IESNU); China, Yunnan Province, Yulong Snowberg, Big ropeway, 4571 m, 29 June 2006, Mingfu Wang Co., 3♂♂(IESNU).

#### Diagnosis.

Arista pubescence, longest hairs shorter than its basal diameter; lower facial margin slightly projecting, situated before frontal angle in profile; legs black; hind tibia with one row of *pv*; inner side of 5th sternite processes with a protrusion.

**Figure 2.**
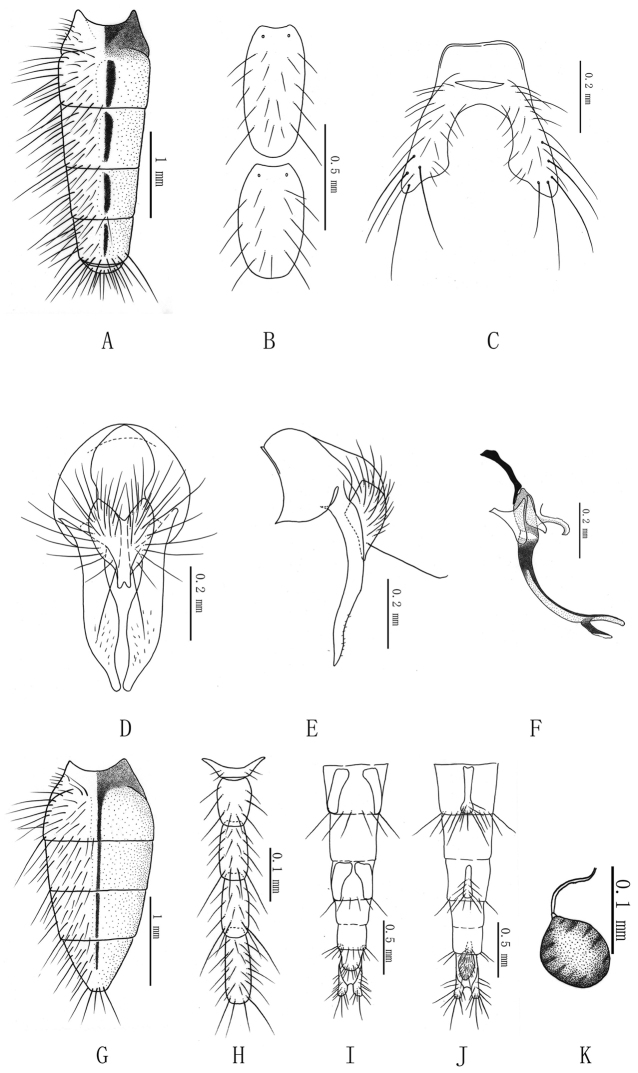
***Delia longiabdomina* sp. n. A** male, abdomen in dorsal view **B** male, 3rd and 4th sternites in ventral view **C** male, 5th sternite in ventral view **D** male, terminalia in posterior view **E** male, terminalia in profile **F** male, aedeagus in profile **G** female, abdomen in dorsal view **H** female, sternites 1 to 5 **I** female, ovipositor in dorsal view **J** female, ovipositor in ventral view **K** female, spermatheca.

#### Description.

**Holotype Male**. Body length 6.5 mm.

*Head*. Eye sparsely with short ciliated; frontal vitta black, with black dust; frontal vitta with a pair of interfrontal setule; frons as wide as anterior ocellus; without orbital setae; fronto-orbital plate with dark grey dust; 6–7 pairs of frontal setae, situated on lower half of frons; parafacial 1.5 times wider than postpedicel; antenna black, postpedicel approx. 1.5–1.8 times longer than broad; arista pubescence, longest hairs shorter than its basal diameter; lower facial margin slightly projecting, situated before frontal angle in profile; gena sparsely with dark grey dust, genal height approx. 1/5 eye height; anterior margin of gena with 1–2 rows of upcurved subvibrissal setulae; para-occipital and postgenal hairs black; proboscis slender, prementum with grey dust, 6.0 times longer than broad; palpus black, subequal to prementum.

*Thorax*. Black in ground colour with dark green lustre and dark grey dust; scutum with three indistinct black vittae, the middle one absent; two rows of hair-like *prst acr* (1 or 2 pairs stronger), only one pair of *post acr* developed, these situated in front of scutellum, *dc* 2+3, *ial* 0+2, with one pair of outer posthumeral setae; notopleural despression bare apart from two strong setae; *pra* subequal to posterior notopleural; scutellum bare on disc centrally and basally; katepisternals 1+2(3).

*Wing*. Base fuscous, basicosta black; costa setulose only basally on ventral surface; costal spine subequal to crossvein r-m; radial node bare, squamae yellowish or white, outer marginal hairs long; lower squama short, approx. 1/2 length of upper; halter brownish yellow.

*Legs*. Entirely black; fore tibia with one medial *p*; mid femur with seta-like *a* row in basal half, *pv* rows complete, becoming shorter apically; mid tibia with two *p*, one long *pd* and 1–2 short *pv*; mid tarsomere 1 with one row of long *pd*, longer than its diameter; hind femur with complete row of *av*, becoming longer apically, with two apical *pv*; hind tibia with 8–10 *av*, 5–6 *ad*, one row of *pd* (4 developed), and one row of *pv*, similar as *Delia platura* (Meigen, 1826); all tarsi shorter than tibiae, claws and pulvilli large.

*Abdomen*. Black, long flat-shaped in dorsal view, with grey dust; all tergites with broad black vittae in middle part, the front margin slightly dark, edge unclear; lateral and post marginal setae long; 6th tergite bare; 1st sternite densely with long setae; 3^rd^ and 4^th^ sternites without dense long setae.

**Female**. Body length 5.0–5.5 mm; frons approx. 2/5 width of head; frontal vitta 2.5 times as wide as fronto-orbital plate; three pairs of orbital setae and three pairs of frontal setae; genal height 1/3 eye height; *dc* 2(3)+3, without outer posthumeral setae; katepisternum 1+1(2); fore tibia with 1–2 *ad*; hind tibia with 4–5 *av*, 5 *ad*, 7–8 *pv*; the other characters as same as male.

#### Remarks.

This new species is similar to *Delia subnigribasis* Fan & Wang in Fan et al., 1981, but differs from it in the following features: male frontal setae 6–7 pairs; *pra* subequal to posterior notopleural seta; inner side of 5th sternite processes broad apically; cercus broad apically, surstylus broad and straight in dorsal view, straight in lateral view, becoming slender apically.

#### Etymology.

The species name is derived from the Latin words “*long*” meaning long, referring to its very long abdomen.

#### Distribution.

China, Yunnan Province (Yulong Snowberg).

### Delia nigerihalteres sp. n.

Taxon classificationAnimaliaDipteraAnthomyiidae

http://zoobank.org/DF2FD042-384E-4D80-9588-F7232528DA76

Figure 3

#### Type material.

***Holotype*.** China, Yunnan Province, Baimang Snowberg, 4100 m, 30 May 2007, Dandan Wang Co., ♂(IESNU). ***Paratype*.** China, same data as holotype, 3♂♂.

#### Diagnosis.

Frons 1.5 times wider than anterior ocellus; without orbital setae; 6–7 pairs of frontal setae; arista ciliated, longest hair longer to 1.5 times basal diameter; anterior margin of gena with 3–4 rows of upcurved subvibrissal setulae; parafacial 1.2 times wider than antenna; prementum approx. 2.5–3.0 times longer than broad; costa setulose only basally on ventral surface; legs black; mid tarsomere 2 without protuberance; all tergites with broad trapeziform spots in centre, posterior marginal setae and lateral setae strong; inner side of 5^th^ sternite processes without protrusion.

**Figure 3.**
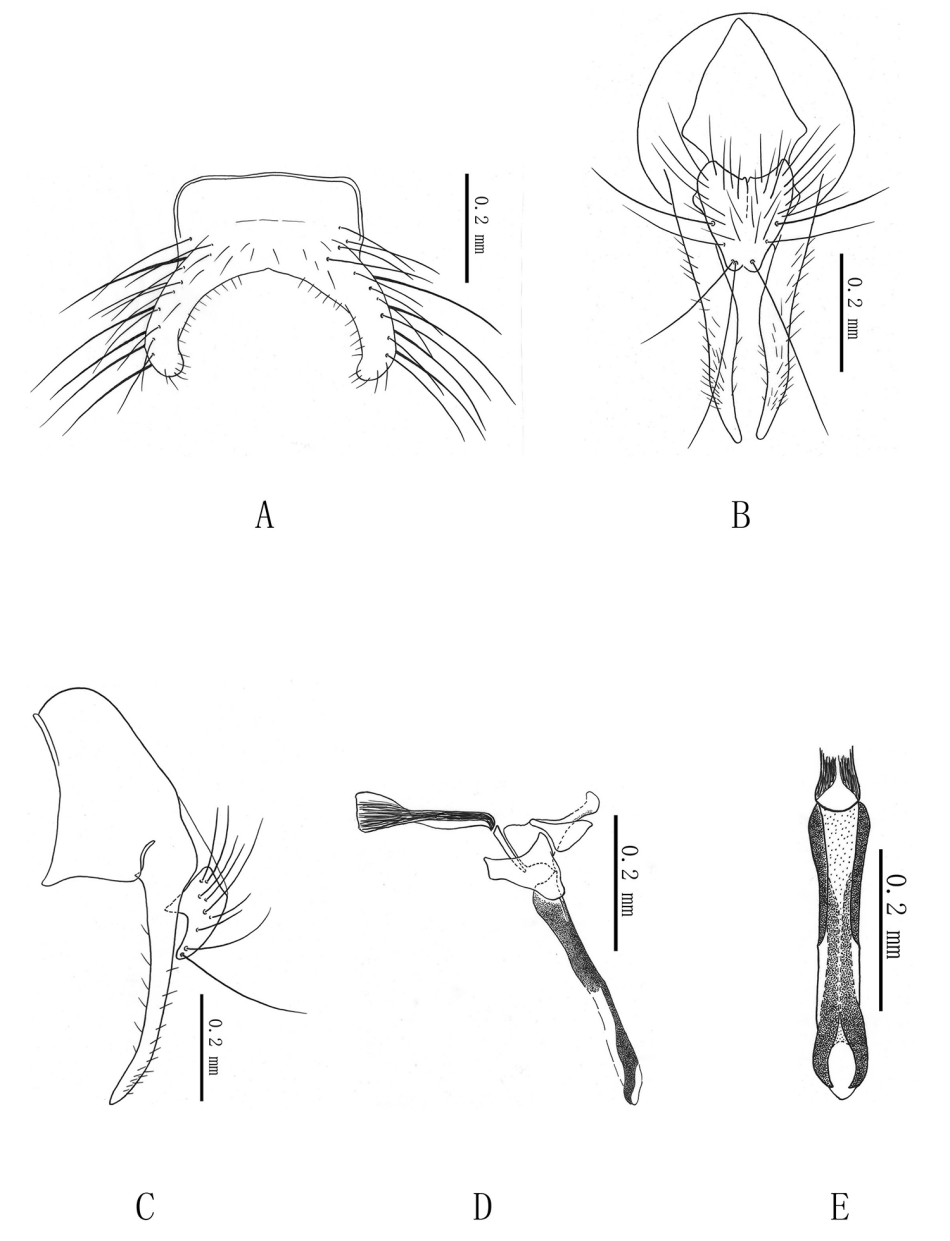
***Delia nigerihalteres* sp. n. A** male, 5^th^ sternite in ventral view; **B.** male, terminalia in posterior view; **C.** male, terminalia in profile; **D.** male, aedeagus in profile; **E.** male, distal part of aedeagus in anterior view.

#### Description.

**Holotype male.** Body length 3.0–3.5 mm.

*Head*. Eye bare; frontal vitta black, 1.5 times as wide as fronto-orbital plate; frons 1.5 times wider than anterior ocellus; frontal vitta with a pair of interfrontal setule; without orbital setae; 6–7 pairs of frontal setae, situated on lower 3/5 of frons; fronto-orbital plate and parafacial with dark grey dust; parafacial 1.2 times wider than antenna; antenna black, postpedicel approx. 1.5–1.8 times longer than broad; arista ciliated, longest hair longer to 1.5 times basal diameter; lower facial margin slightly projecting, vibrissal angle situated before frontal angle in profile; genal height approx. 1/5 eye height; anterior margin of gena with 3–4 rows of upcurved subvibrissal setulae; prementum shiny, approx. 2.5–3.0 times longer than broad; palpus black, slightly shorter than prementum.

*Thorax*. Black in ground colour with brownish grey dust; scutum with three black vittae; two rows of hair-like *prst acr* (the second pair stronger), only one pair of *post acr* developed, these situated in front of scutellum, *dc* 2+3, *ial* 0+2; notopleural despression bare apart from two strong setae; *pra* subequal to the posterior notopleural; scutellum bare on disc centrally and basally; katepisternals 1+2.

*Wing*. Base and veins fuscous, basicosta black; costa setulose only basally on ventral surface; costal spine absent; radial node bare, squamae brown; lower squama approx. 1/2 length of upper; halter black.

*Legs*. Entirely black; fore tibia with one medial *p*; mid femur with a complete row of *av*, becoming shorter apically, a complete row of *pv*, becoming longer medially; mid tibia with 2–3 *pd* and two *pv*; mid tarsomere 1 with row of long *pd*, subequal to the length of its diameter; hind femur with complete row of *av* and *pv*, becoming longer mediately; hind tibia with five *av*, 5–6 *ad*, 2–3 *pd*, and 8–9 *pv*; all tarsi shorter than tibiae, claws slightly longer than pulvilli, pulvilli approx. 1/2 length of tarsomere 5.

*Abdomen*. Black, flat cone-shaped in dorsal view; all tergites with broad trapeziform spots in centre, posterior marginal setae and lateral setae strong; 6^th^ tergite bare; 1^st^ sternite with fine hairs.

**Female.** Unknown.

#### Remarks.

This new species is similar to *Delia quadrilateralis* Fan & Zhong in Fan et al., 1981, but differs from it in the following features: male frontal vitta 1.5 times as wide as fronto-orbital plate; frons 1.5 times wider than anterior ocellus; basicosta black; costal spine absent; squamae brown; halter black; mid tarsomere 1 with row of long *pd*, subequal to the length of its diameter.

#### Etymology.

The specific name is from the Latin word “*niger*”, black, “*halter*”, halter, referring to its black halter.

#### Distribution.

China, Yunnan Province (Baimang Snowberg).

### Delia tuberisurstyla sp. n.

Taxon classificationAnimaliaDipteraAnthomyiidae

http://zoobank.org/CD4AC08A-4682-4CA8-BD74-B96336BB2783

Figure 4

#### Type material.

***Holotype*.** China, Yunnan Province, Yulong Snowberg, Big ropeway, 4571 m, 29 June 2006, Ming-Fu Wang Co., ♂(IESNU). ***Paratype.*** China, same data as holotype, 1 ♂.

#### Diagnosis.

Arista pubescence, longest hairs shorter than its basal diameter; lower facial margin slightly projecting, with anterior margin of frons in same vertical plane; legs black; hind tibia with two rows of *pv*; inner side of 5^th^ sternite processes with a protrusion.

**Figure 4.**
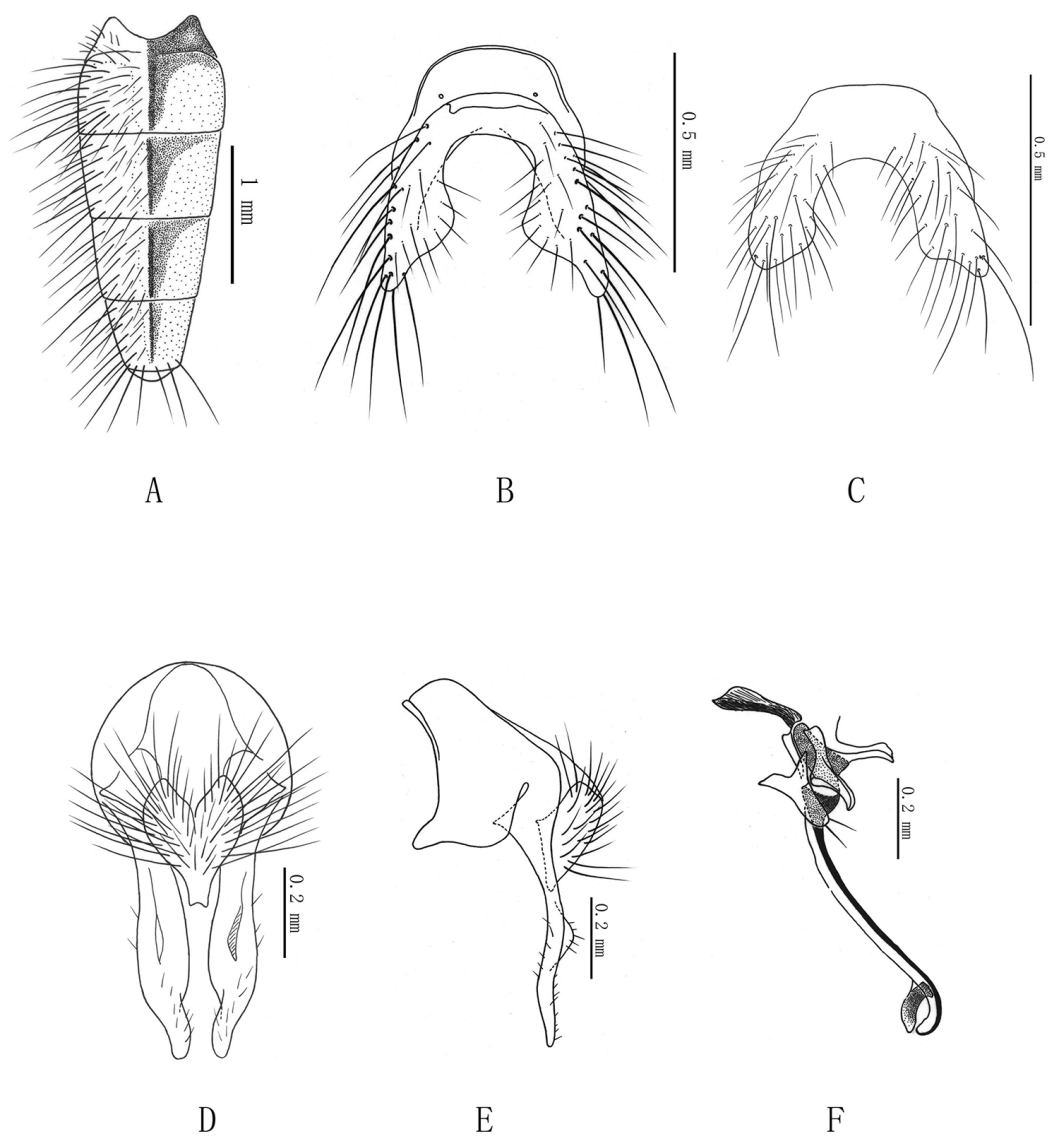
***Delia tuberisurstyla* sp. n. A** male, abdomen in dorsal view; **B** male, 5^th^ sternite in ventral view, in holotype; **C** male, 5^th^ sternite in ventral view, in paratype **D** male, terminalia in posterior view **E** male, terminalia in profile **F** male, aedeagus in profile.

#### Description.

**Holotype male**. Body length 5.5–6.0 mm.

*Head*. Eye with sparse and short ciliae; frontal vitta black, with black dust; frontal vitta with a pair of interfrontal setule; frons as wide as anterior ocellus; without orbital setae; fronto-orbital plate, parafacial and gena with dark grey dust; 7–8 pairs of frontal setae, situated on lower half of frons; parafacial 1.4 times wider than postpedicel; antenna black, postpedicel 1.5 times longer than broad; arista pubescence, longest hairs shorter than its basal diameter; lower facial margin slightly projecting, with anterior margin of frons in same vertical plane; genal height approx. 2/7 eye height; anterior margin of gena with two rows of upcurved subvibrissal setulae; para-occipital and postgenal hairs black; proboscis slender, prementum with grey dust, approx. 6.0–7.0 times longer than broad; palpus black, subequal to prementum.

*Thorax*. Black in ground colour with dark grey dust; scutum with three indistinct black vittae; two rows of hair-like *prst acr* (1 or 2 pairs stronger), only one pair of *post acr* developed, these situated in front of scutellum, *dc* 2+3, *ial* 0+2; notopleural despression bare apart from two strong setae; *pra* 1.2 times longer than posterior notopleural seta; scutellum bare on disc centrally and basally; katepisternals 1+2.

*Wing*. Base fuscous, basicosta black; costa setulose only basally on ventral surface; costal spine absent; radial node bare, squamae yellowish; lower squama short, approx. 1/3 length of upper; halter brown-yellow.

*Legs*. Entirely black; fore tibia with 1(0) medial *p*; mid femur with seta-like *a* row in basal half, *pv* rows complete; mid tibia with one *pd* and two *pv*; mid tarsomere 1 with one row of long *ad*, more than 1.5 times longer than its diameter; hind femur with complete row of *av*, becoming longer apically; hind tibia with 9–10 *av*, 5–6 *ad*, one row of *pd* (3–4 developed), and two rows of *pv*, slightly pectinated; all tarsi shorter than tibiae, claws and pulvilli large, slightly shorter than tarsomere 5.

*Abdomen*. Black, long cone-shaped in dorsal view, with grey or brownish grey dust; all tergites with hair-like setae in middle part, setae becoming longer towards lateral margin, mid black vitta expand in middle part, near rhombic; 6^th^ tergite bare; 1^st^ sternite dense with long fringes.

**Female.** Unknown.

#### Remarks.

This new species is similar to *Delia subnigribasis* Fan & Wang in Fan et al., 1981, but differs from it in the following features: male frontal setae 7–8 pairs; *pra* 1.2 times longer than posterior notopleural seta; basicosta black; mid tarsomere 1 with one row of long *ad*, more than 1.5 times longer than its diameter.

#### Etymology.

The species name is derived from the Latin words “*tuber*”, tuber, referring to the middle part of surstylus with a sheet-shaped tuber in dorsal view.

#### Distribution.

China, Yunnan Province (Yulong Snowberg).

### Distribution of the known species from Yunnan Province

***Delia absidata* Xue & Du, 2008: 113–122**

**Distribution**. China: Yunnan Province, Shangri-la, Bitahai (type loc.).

***Delia antiqua* (Meigen, 1826): 166**

**Distribution**. Worldwide distribution (type loc. Germany): China (Heilongjiang, Jilin, Liaoning, Inner Mongolia, Gansu, Qinghai, Heibei, Beijing, Shanxi, Shandong, Shanghai, Sichuan, Yunnan).

***Delia aurosialata* Fan, 1993 in Fan & Zheng: 1128**

**Distribution**. China: Yunnan Province, Yunlong, Mt. Zhiben (type loc.).

***Delia bracata* (Rondani, 1866): 183**

**Distribution**. China (Yunnan, Tibet); India; Iran; Israel; Lebanon; Spain; France; Greece; Hungary; Italy (type loc.); Poland.

***Delia echinata* (Séguy, 1923): 360**

**Distribution**. China (Sichuan, Yunnan, Tibet); Japan; North Korea ; India; Israel; Russia; Austria; Czech Republic; Slovakia; Germany; France (type loc.); England; Greece; Italy; Iceland; Roumania; Sweden; Finland; Yugoslavia.

***Delia floralis* (Fallén, 1824): 71**

**Distribution**. China (Heilongjiang, Liaoning, Inner Mongolia, Qinghai, Xinjiang, Hebei, Shanxi, Yunnan); Japan; North Korea; Russia; Czech Republic; Slovakia; Germany; France; England; Denmark; Spain; Hungary; Norway; Sweden (type loc.); Finland.

***Delia linearis* (Stein, 1898): 219**

**Distribution**. China (Heilongjiang, Jilin, Xinjiang, Shanxi, Yunnan); Japan; Czech Republic; Germany (type loc.); France; England; Poland; Sweden; Finland; Yugoslavia; Estonia; Latvia; Lithuania; Russia; White Russia; White Russia; Nearctic region.

***Delia longitheca* Suwa, 1974: 160**

**Distribution**. China (Heilongjiang, Liaoning, Henan, Shanxi, Shaanxi, Sichuan, Guizhou, Yunnan); Japan (type loc.); North Korea; Russia.

***Delia partivitra* Fan, 1993 in Fan & Zheng: 1131**

**Distribution**. China: Yunnan Province, Lijiang, Mt. Yulong (type loc.).

***Delia platura* (Meigen, 1826): 171**

**Distribution**. Worldwide distribution (type loc. Germany).

***Delia sclerostylata* Fan, 1993 in Fan & Zheng: 1134**

**Distribution**. China: Yunnan Province, Lushui, Yaojiaping (type loc.).

***Delia subinterflua* Xue & Du, 2008: 113–122**

**Distribution**. China: Yunnan Province, Mt. Yulong, Big ropeway (type loc.); Sichuan, Mt. Balang.

## Supplementary Material

XML Treatment for Delia dentiaedeagus

XML Treatment for Delia longiabdomina

XML Treatment for Delia nigerihalteres

XML Treatment for Delia tuberisurstyla
